# Gout flares, serum urate and seasonality: a descriptive cohort study

**DOI:** 10.1007/s10067-025-07898-8

**Published:** 2026-01-06

**Authors:** Samuel Finnikin, Christian D. Mallen, Edward Roddy

**Affiliations:** 1 Department of Applied Health Sciences, University of Birmingham, Birmingham, B15 2TT UK; 2 Primary Care Centre Versus Arthritis, School of Medicine, Keele University, Keele, UK; 3 Haywood Academic Rheumatology Centre, Midlands Partnership University NHS Foundation Trust, Stoke-On-Trent, UK

**Keywords:** Gout, Seasons, Uric acid

## Abstract

**Aims:**

To investigate the relationship between serum urate levels and gout flares and how these vary at different times of the year.

**Methods:**

A cohort of people with incident gout was established using a large UK primary care database (Clinical Practice Research Database). Clinician-recorded gout flares and serum urate (SU) measurements were identified and described using joinpoint linear regression modelling. The relationship between SU level, flare frequency, month of the year and mean monthly temperatures was explored and correlations tested using Pearson correlation coefficients.

**Results:**

249,157 individuals (mean follow-up 6.7 years) experienced 417,101 flares and had 341,457 SU measurements (mean SU 437 µmol/L, standard deviation (SD) 106 µmol/L). SU levels peaked the day before a flare (487 µmol/L). Mean SU in the year preceding a flare was 474 µmol/L compared with 432 µmol/L in the year post-flare. SU levels did not near pre-flare levels in the year following a flare.

Flares were most frequent, and SU was highest, in the summer months (June to August). The correlation co-efficient between flares and months of the year was 0.94, whereas the correlation with temperature was less strong (0.70).

**Conclusions:**

SU measurement in the year following a gout flare is not indicative of the peak (pre-flare) SU levels that an individual may have experienced. Clinicians should consider this when considering SU measurements in the diagnosis of gout. Patients may find it helpful to be informed of the seasonality of gout flares and advised to take extra caution to reduce the risk of flares during summer months.

**Table:** 

**Keypoints** • *Serum urate (SU) levels drop precipitously during gout flares and remain low for several months.* • *Correlations exist between flare rate and summer months, and with seasonal variation in SU levels.* • *Clinicians should be aware of how gout flares affect SU when interpreting SU levels.*

**Supplementary Information:**

The online version contains supplementary material available at 10.1007/s10067-025-07898-8.

## Introduction

Gout is the most common inflammatory arthritis with a global incidence of 0.58–2.89 per 1000 person years and a prevalence in the UK of 3.2% [1, 2]. Both the prevalence and incidence of gout are rising worldwide [1], and, in contrast to other inflammatory arthritides, gout is almost exclusively managed in primary care.

Gout is characterised by acute inflammatory flares caused by crystallisation of monosodium urate (MSU) within the joint [3, 4]. The risk of gout flares increases with a higher serum urate level (SU) [5–7] and is reduced by urate-lowering therapies (ULT) such as allopurinol and febuxostat [8]. Other factors that contribute to the formation of MSU crystals include lower temperature, lower pH and physical shock [9]. It is recommended that SU is measured to aid diagnosis of gout, but it is noted that SU may be reduced during a flare [10, 11] so testing at least 14 days after a flare is recommended in order to avoid diagnostic confusion caused by a temporarily reduced SU [12, 13].

Gout flares show seasonal variation; with the highest flare occurrence reported in spring in studies from the USA, Korea and Italy [14–16], and summer in the UK [17]. The exact mechanism underlying seasonality variation is unknown but it has been postulated that it could be due to deviations of temperatures from the mean rather than absolute temperatures that influence gout flares [18]. It is plausible that hotter weather leads to relative dehydration and therefore an increase in SU which may contribute to the seasonality of gout flares [19]. However, when the seasonality of serum urate levels has been studied the relationship is complex, with no clear variation in SU seen in spring or summer [20]. It could also be that the seasonality of gout flares is influenced by other factors such as the consumption of food or drinks that could provoke a flare (such as alcohol), trauma to joints, or the solubility of urate (which is lower at lower temperatures) [21].

Screening or routine measurement of SU in healthy individuals is not standard practice in the UK, but SU should be measured in patients with a suspected diagnosis of gout or when initiating or monitoring ULT [12]. However, SU measurement is known to be suboptimal with no SU measurements being undertaken in around 40% of patients with gout [5, 22].

The relationships between gout flares, serum urate and the seasons have, so far, been established using relatively small cohorts and timeframes. Furthermore, little is known about SU levels prior to gout flares. The aims of this study were to investigate the relationship between serum urate and gout flares in people not treated with ULT, how these vary with the seasons and temperature, as well as how rates of testing of serum urate have varied over time in people with gout in the UK.

## Methods

This retrospective cohort study comprised patients with incident gout in the Clinical Practice Research Datalink (CPRD) AURUM database [23]. CPRD AURUM contains anonymised primary care records including demographics, coded diagnoses and prescribing data for over 16 million patients from 1771 practices (around 23.9% of the UK population) and, with over 98% of the UK population registered with a GP [24], it is broadly representative of the demographics of the UK population.

The data extraction and cohort selection according to study design were facilitated using the data extraction for epidemiological research (Dexter) tool [25]. Patients aged 20 years and over from practices opting into CPRD AURUM were included in the cohort if they had a first, coded diagnosis of gout (or gout-related code such as tophi) between 1 st January 2010 and 31 st December 2023. Clinical codes used are provided in supplementary table S1. Previous research has validated the coding of gout in UK electronic medical records to an acceptable level (90% accuracy when combined with urate levels and/or prescribing data) and even when the relatively low levels of serum urate testing are considered, the PPV of a primary care diagnosis of gout in the UK has been found to be high at 88.6% [22, 26].

Patients were eligible for inclusion from the study start date or the earliest of either the practice standardisation date (the date at which the practice data is deemed to be of research quality, based on CPRD algorithm) or the date the patient registered with the practice plus 1 year (to allow time for records to be transferred); until the earliest of study end date, end of practice data or death. The date of the first incident diagnosis of gout was defined as the index date and patients were excluded if they left the database within 180 days of this date to allow for a minimum of 180 days follow-up. Patients were also excluded if they had a prior diagnosis of gout (or related code) or had a prescription of ULT (allopurinol or febuxostat at any dose) prior to, or within 30 days subsequent from, the index date. This latter criterion was to identify and exclude patients started on colchicine or NSAIDs prophylactically because of ULT initiation rather than for flare treatment. In order to ensure that none of the cohort were taking ULT during the observation period, prescription of ULT was a censoring event and any co-prescription of NSAID or colchicine on ULT initiation was not counted as a flare. All flares following the index flare are termed ‘subsequent flares’.

All subsequent flares were identified according to criteria previously used [5, 27]. A gout flare was defined as follows: either (1) a recorded prescription of colchicine or (2) a healthcare visit recording a gout code (from any source including letters received and coded by practices following Emergency Department or hospital encounters) together with at least one of the following treatments within 7 days of the code: intra-articular aspiration, intra-articular injection or corticosteroid, prescription of an oral NSAID or oral corticosteroids. Ascertainment of flares was performed recursively. Every new follow-up period following a flare included a grace period of 30 days from the date of the flare detected (to allow for full remission of that flare). Covariates included were sex, age and ethnicity. A ‘missing’ category was used for missing covariate data as these were assumed not to be missing at random. All SU measurements coded after the index event (diagnosis of gout) up until the end of follow-up were extracted along with the latest SU prior to diagnosis where available. Biologically implausible values were identified and excluded as were entries where the SU was recorded as zero.

### Statistical analysis

The cohort was described according to demographic variables, number of flares, ULT treatment and SU levels. Serum urate measurements post-diagnosis were described. For individuals with one or more SU measurements, each SU result was associated with the closest flare in time (where available) before or after that result. The number of days before or after the nearest flare was then calculated. The association between SU and flares was illustrated graphically. Given that an individual cohort member may have multiple SU measurements and flares over time, a subgroup analysis was undertaken of only individuals who had one subsequent flare during follow-up. A further subgroup analysis was undertaken on individuals who only had one subsequent flare during follow-up and had a SU level within one year before *and* one year after the flare. For each of these three groups, a joinpoint linear regression model analysis was undertaken using Joinpoint Regression Program, Version 5.3.0.0 [28]. The joinpoint model was constrained to a minimum of 0 and a maximum of 4 joinpoints.

Seasonality of flares was illustrated by establishing the mean flares/day for each calendar month. The relative risk of flares for each month was calculated with reference to the lowest monthly rate. The mean serum urate level for each month was calculated and illustrated alongside flare frequency and the correlation between flare rate and month was tested using the Pearson correlation coefficient. The flare rate was also correlated with average monthly temperatures for the UK according from the national meteorological service (the MET office) [29]. The rate of SU testing was established for each year of the study to illustrate the trend in testing over time.

The study protocol was reviewed and approved by CPRD (Protocol 24_004339).

## Results

**Fig. 1:**
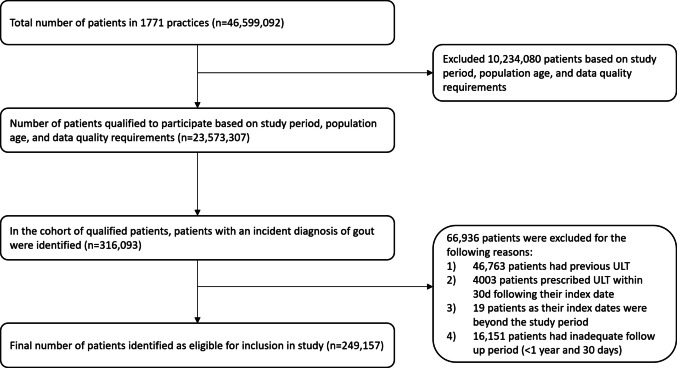
Consort diagram

The cohort comprised 249,157 individuals from 1771 separate practices (see STROBE diagram Fig. 1) with a mean follow-up period of 6.7 years (standard deviation (SD) 3.6 years). Mean age (SD) was 61.7 (15.5) years and the majority were male (74.6%) and of white ethnicity (81.3%) (Table 1). There were 417,101 flares recorded and at least one subsequent flare was identified for 191,064 (76.7%) individuals.

**Table 1:** Characteristics of the cohort

		*n* (%)
Total population		249,157 (100)
Age category	20–49	63,480 (25.5)
	50–59	49,274 (19.8)
	60–69	53,625 (21.5)
	70–79	50,066 (20.1)
	80 +	32,712 (13.1)
Sex	Male	184,843 (74.6)
	Female	63,311 (25.4)
	Missing	3 (0.0)
Ethnicity	White	202,515 (81.3)
	Black	6106 (2.5)
	Asian	13,698 (5.5)
	Mixed	797 (0.3)
	Other	1896 (0.8)
	Missing	24,145 (9.7)
Flares post index	0	58,093 (23.3)
	1	100,989 (40.53)
	2	40,884 (16.4)
	3	19,505 (7.8)
	4	10,387 (4.2)
	5 +	19,299 (7.7)
ULT started^a^	Yes	90,359 (36.3)
Patients with serum urate level^b^	< 30 d pre-index	62,782 (25.2)
	≥ 30 pre-index index	53,188 (21.4)
	Post index	162,453 (65.2)
	None ever	49,248 (19.8)

^a^Censoring event

^b^Patients may fall into > 1 category

### Serum urate measurements

**Fig. 2:**
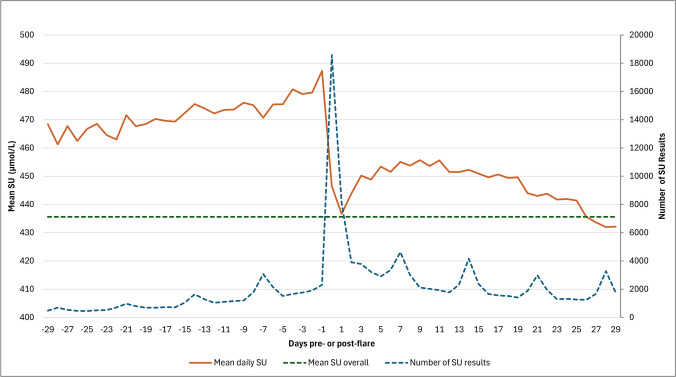
Mean serum urate and number of serum urate results and the number of days pre- or post-flare

There were 341,457 SU measurements post-index date (diagnosis) in 162,453 individuals with a mean SU of 435.6 µmol/L (95% confidence interval (95% CI) 435.2–435.9µmol/L). For people who experienced at least one subsequent flare, the mean SU was 437.9 µmol/L (437.5–438.2 µmol/L) from 286,086 measurements, compared with a mean of 423.9 µmol/L (423.0–424.7 µmol/L) from 55,371 measurements in those who did not flare during the follow-up period. The highest daily mean SU level was 487.3 µmol/L (482.9–491.7 µmol/L) observed on the day before a flare (Fig. 2). Mean levels dropped off post-flare to a low of 432.0 µmol/L (424.3–425.2 µmol/L) 29 days following a flare. It took 26 days post-flare for the mean daily SU to drop to the overall SU mean.

Considerably more SU results were recorded on the day of a flare compared with any other day with 18,579 results on day 0 compared with between 469 (day −25) and 4632 (day 7). There appeared to be weekly peaks in the frequency of SU results post-flare and, to a lesser extent, pre-flare. 62,782 (25.2%) patients had an SU measured in the 30 days prior to the date of diagnosis suggesting SU was used in the diagnostic pathway for these patients. Overall, 49,248 (19.8%) of the cohort did not have an SU recorded prior to gout diagnosis or at any stage during follow-up. The overall rate of SU testing was relatively consistent during the cohort with an upward trend in testing rates following a low in 2020 (supplementary figure S1).

**Fig. 3:**
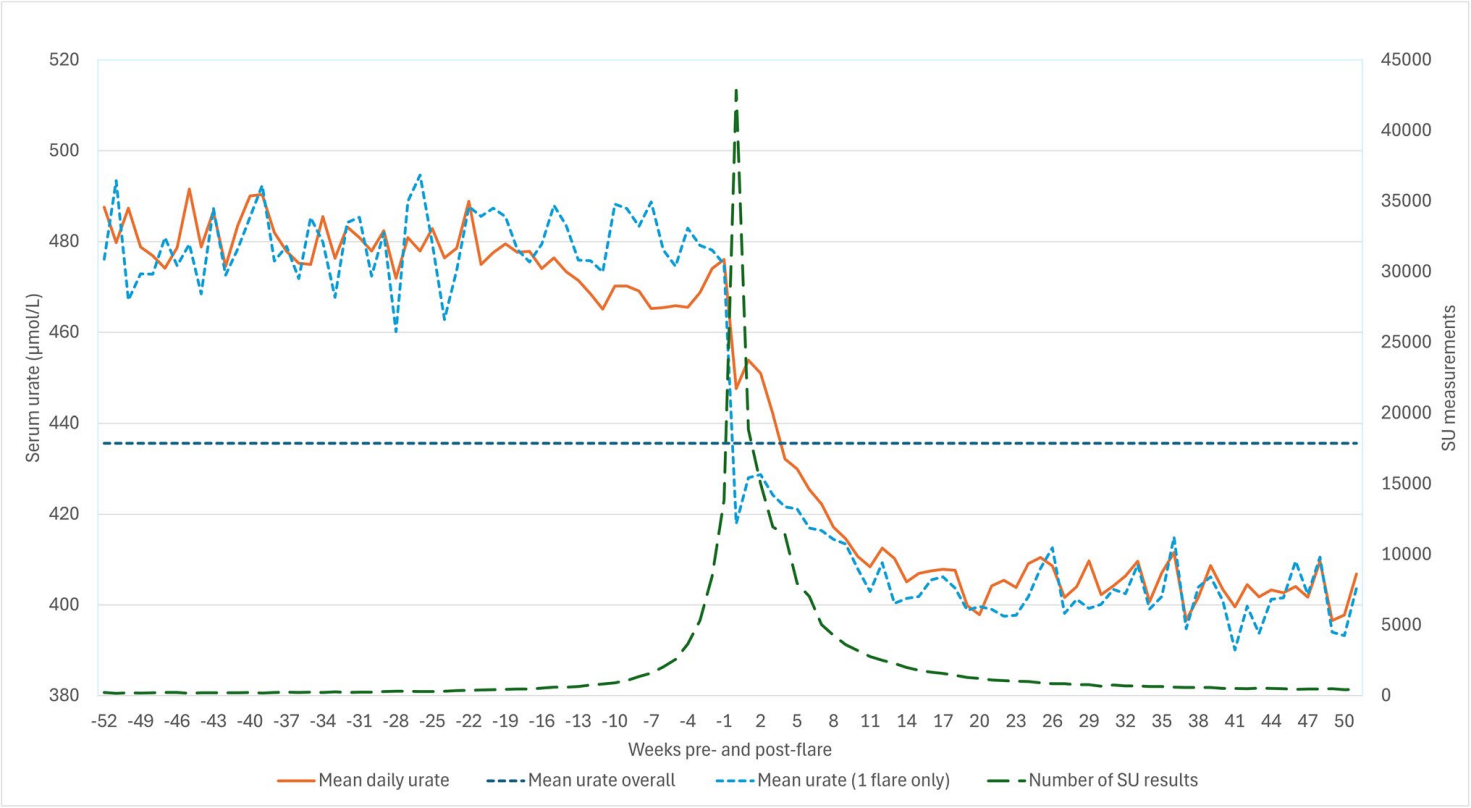
Weekly mean serum urate and number of serum urate results and the number of weeks pre- or post-flare

Extending the period of observation pre- and post-flare, we observed that SU remained significantly lower for 1-year post-flare than it was pre-flare (Fig. 3). The mean SU in the year preceding a flare was 473.7 µmol/L (472.8–474.5 µmol/L) from 56,860 SU measurements compared with 432.2 µmol/L (431.7–432.7 µmol/L) from 168,307 SU measurements in the year post flare. Restricting the analysis to patients who only experienced one flare during follow-up shows a similar pattern (Fig. 3) and difference in means (479.0 µmol/L (477.2–480.8 µmol/L) from 11,293 pre-flare SU measurements and 414.9 µmol/L (414.1–415.7 µmol/L) from 63,368 post-flare SU measurements).

**Fig. 4:**
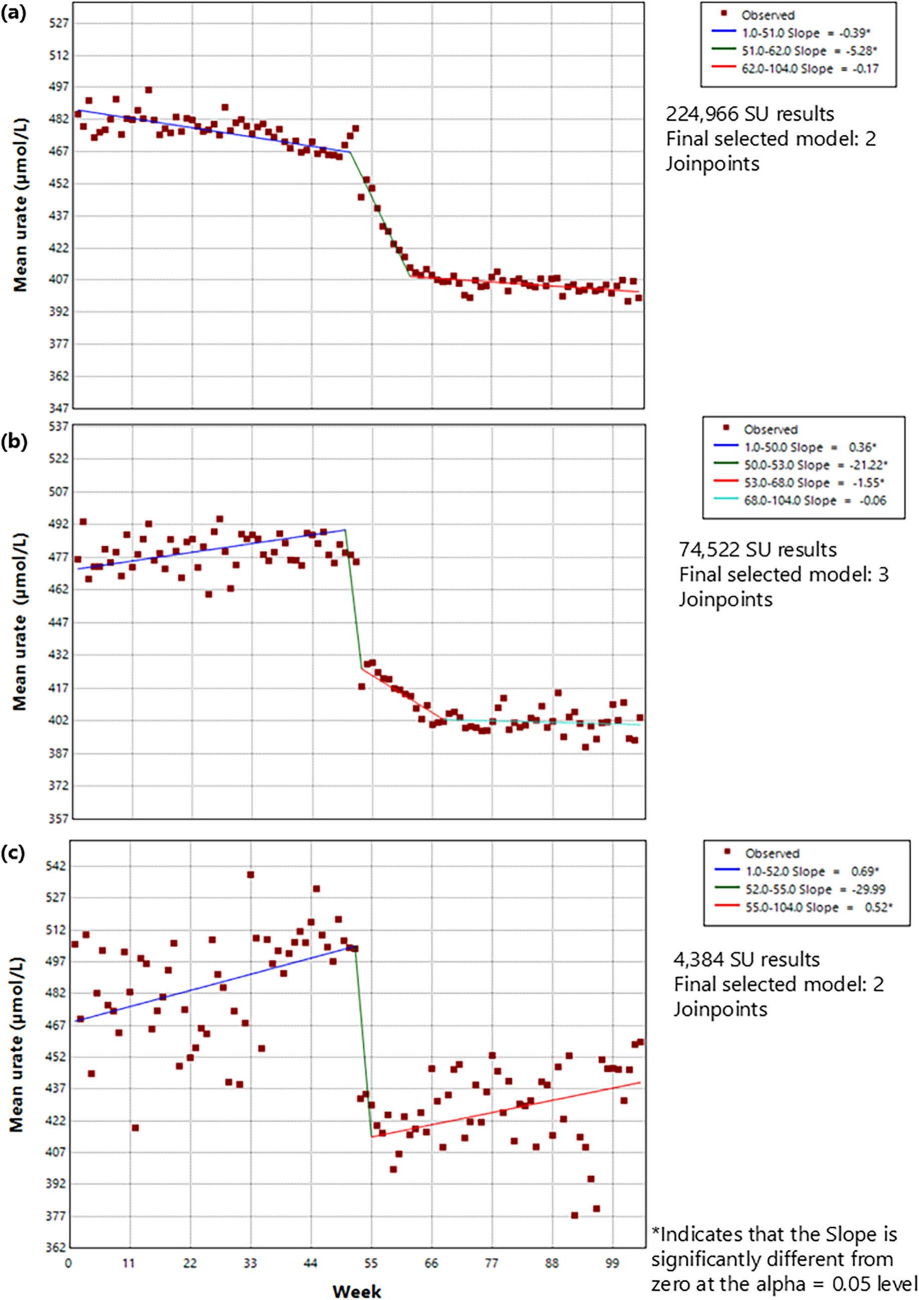
Joinpoint models for mean weekly SU in the year pre- and post- flare for **a** all; **b** individuals with only one subsequent flare during follow up; **c** individuals with one subsequent flare during follow up and a pre- and post-flare SU measurement

The final selected joinpoint model for the whole cohort (model a) comprised 2 joinpoints (Fig. 4, supplementary figure S2). These correspond to a pre-flare trajectory, a post-flare trajectory and a ‘during flare’ period of 9 weeks. Model (b), for patients with only one flare during follow-up, has 3 joinpoints as the final selected model suggesting a rapid decrease in the first 5 weeks of the flare period and then a slower drop-off for a further 15 weeks before an almost constant SU in the subsequent time period (weakly SU change of − 0.06 µmol/L).

The smallest subgroup consisted of individuals who had just one flare during follow-up and who had a recorded SU in the year before and the year after the flare (model (c)). As with model (a), the final selected model for this subgroup had 2 joinpoints but with a shorter (3 week) ‘during-flare’ period. Using the rates of change in SU in this model, it would take an estimated 225 weeks for the SU to increase from a post-flare low to the pre-flare high, or, in the opposite direction, 130 weeks for the pre-flare high to have developed from a previous post-flare low.

### Seasonality

**Fig. 5:**
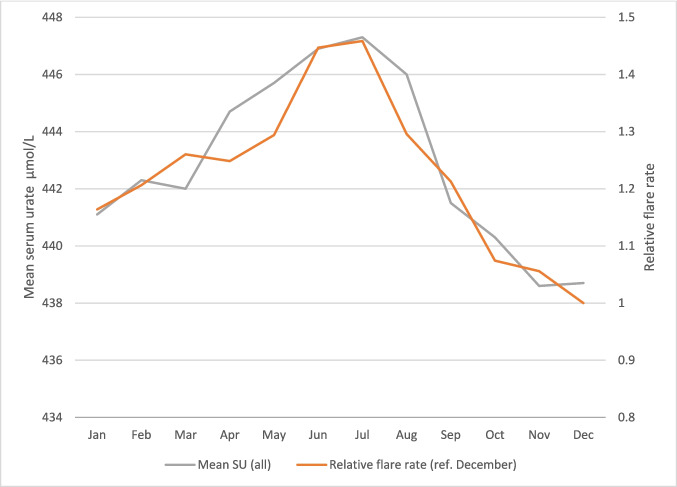
The relative rate of flares and mean serum urate (all recorded) by calendar month

Mean SU levels were highest from April to August (Fig. 5). July had the highest mean SU (447.3, 446.4–448.3 µmol/L) and November the lowest (438.6, 437.4–439.7 µmol/L). Similarly, flares were most frequent during April to August and lowest in the winter months with the rate of flares being 1.46 times higher in July than December (Fig. 5). The relationship between monthly mean SU and relative flare rate appeared linear (supplementary figure S3) (Pearson correlation coefficient 0.94). MET office temperature data over this time period shows the highest average temperatures were in July and August (15.6 °C and 15.1 °C respectively) and the lowest average temperatures in December, January and February (4.6 °C, 3.9 °C and 4.3 °C, respectively) (supplementary data S1) [29]. The Pearson correlation coefficient between mean monthly temperatures and flare rate was 0.70.

## Discussion

In this cohort of people with gout, we investigated the relationship between gout flares and SU levels. The large cohort size allowed new insights into SU levels in the days to weeks preceding a gout flare that is otherwise difficult to study given the unpredictable nature of flares. In the three different subgroups analysed, mean SU levels were significantly lower post-flare than pre-flare. There was a precipitous reduction in SU at the time of the flare and, although the SU levels rose after the flare, they did not return to the pre-flare level for as long as a year after the flare. This observation could not be due to ULT prescribing since ULT is almost exclusively prescribed in primary care so would be evident in the CPRD dataset and none of the cohort were prescribed ULT at any stage.

We have demonstrated a clear relationship between flare rate and the month of the year, with the highest flare rate observed during the summer months although this relationship did not appear to be wholly explained by average temperatures. There was a corresponding seasonal pattern in SU levels with a peak mean SU observed in July. There was a good correlation between mean monthly SU levels and flare rate. Finally, the majority of SU testing was done at the time of flares and the annual rate of SU testing was relatively static during the study period with a slight dip during the years affected by the covid pandemic. The weekly periodicity in SU testing following a flare may be due to a clinician heuristic of measuring SU a set number of weeks following a flare (‘get your bloods done in 2 weeks time’) as well as an artefact of transposing a 5-day working pattern onto a 7-day calendar.

### Comparisons with existing literature

Several small secondary care cohorts have examined SU levels both during and in between flares in patients with gout not taking ULT [10, 11, 30]. These have shown similar mean SU levels during flares to levels we observed but higher interval levels. This may be explained by secondary care cohorts consisting of more severely affected patients whereas our primary care cohort includes people with less severe disease and associated lower SU levels. A larger cohort, established to investigate the use of etoricoxib for gout flares, found similar SU levels around the time of flare (428 µmol/L) with a slight increase to 434 µmol/L 8 days later [31].

The summer peak in UK gout incidence has been previously reported [17], and this could be explained by the hotter weather causing relative dehydration and higher SU concentrations precipitating a flare. It may also be that greater alcohol consumption during the summer months [32] contributes to the observed peak given the association with alcohol consumption and gout flares for which a number of mechanisms have been proposed including temporary lactic acidaemia leading to reduced renal urate excretion and exacerbation of hyperuricaemia [33, 34]. Higher SU levels during the summer months correlated well with the flare rate and could potentially be due to an increase in dietary purine consumption. Other studies have found a seasonal pattern to SU levels with increasing SU in hotter months [20, 35–37]. A meta-analysis of seasonal variation in gout flares by month reported a similar pattern to the one we observed with gout rates increasing from March to a peak in July with a steep drop off over August and September [38]. However, individual studies around the world have found different peak months [14–16, 20, 39]. It has been postulated that it is not high absolute temperatures that predisposes to gout flares but rather high day-to-day variability in temperature and humidity [16] which may explain the poor correlation between mean temperatures and gout flare rate that we observed. It could also be that the reduced solubility of urate at lower temperatures [40] affect the relationship between seasonal temperature variation and gout flares.

### Strengths and limitations

By using a very large dataset of routinely collected and longitudinal data over a long period of time we have been able to illustrate the way in which SU changes around the time of a gout flare in more detail than has previously been described. Moreover, using a primary care dataset increases the generalisability of the findings given that most gout patients are managed in this setting. Further strengths include using established methods of flare ascertainment using routinely collected data and joinpoint regression analysis.

Using routinely collected data risks both over- and under-ascertainment of gout flares. Some recorded clinical contacts for gout may have been erroneously identified as a flare, and some patients will self-managing flares without consulting their practice. Additionally, as gout is a primarily clinical diagnosis in the UK [12] and there are common symptoms with other diagnoses such as acute calcium pyrophosphate crystal synovitis (CPPD), it is possible that some people are mis-diagnosed. However, given the relatively low prevalence of CPPD (0.5% [41]) and high positive predictive value for a primary care diagnosis of gout [22], we feel the impact of misdiagnosis on the findings would be small. Interestingly, CPPD flares do not appear to demonstrate seasonal variation [14, 15].

It is also acknowledged that the analysis treats gout as a homogenous pathology but in reality, there is evidence that people experience different disease trajectories which we did not account for [42]. Importantly, our data cannot explain why we found a greater reduction in the SU level after a flare than might have been expected. Some of the reduction is likely due to the pathophysiology of gout including increased urinary excretion of uric acid during a gout flare [11, 30], but it is possible that patients make changes after consulting for a gout flare resulting in further reductions in SU levels. For example, following advice from their GP, people may make medication changes (e.g. stopping or reducing diuretics) or lifestyle changes (e.g. reducing alcohol intake or maintaining higher levels of hydration) with the intention of reducing their SU and the risk of subsequent flares.

The temperatures used for analysis were UK mean temperatures over the duration of the cohort whereas the practices contributing to this study were all based in England. UK temperatures are, therefore, only an approximation of temperatures patients will have experienced and there will have been variation depending on exact location and from year to year. This makes the relationship between flares and SU levels with temperatures less robust.

## Conclusions

These data provide new insight into how SU levels change over time and challenge existing understanding regarding the speed of rebound of SU levels post-flare. It is apparent that an SU measurement in the year following a gout flare is not indicative of the peak (pre-flare) SU levels that an individual may have experienced, a finding that warrants further investigation. Clinicians should exercise caution when using SU in diagnostic reasoning and lower SU levels should not be used to discount gout in the presence of typical clinical features. However, SU levels post-flare have an important role in a treatment-to-target management strategy should ULT be initiated. Patients may find it interesting to be informed of the seasonality of gout flares and advised to take extra caution to reduce the risk of flares during summer months such as reducing alcohol consumption and staying well hydrated.

## Supplementary Information

Below is the link to the electronic supplementary material.

ESM 1 (DOCX 1.09 MB)

## Data Availability

Primary data is not available through the authors due to data sharing restrictions but is available to researchers through CPRD (subject to approvals). Code lists used in data extraction and processing are available on request from the corresponding author.
