# Supplementary material for: Gout flares, serum urate and seasonality: a descriptive cohort study
Source: Clin Rheumatol. 2026 Jan 6;45(2):1439–48. doi: 10.1007/s10067-025-07898-8 (PMC12858545; doi:10.1007/s10067-025-07898-8)

Gout flares, serum urate and seasonality: a descriptive cohort study

Supplementary materials

Contents:

Supplementary data S1: MET office data for mean UK temperatures between 2010 and 2023 (inc)

Supplementary table S1: Gout diagnostic codes

Supplementary figure S1: Annual rate of urate testing for each year of the study

Supplementary figure S2: Jointpoint models (detail)

Supplementary figure S3: Relationship between mean monthly SU and flare rate

Supplementary data S1: MET office data for mean UK temperatures between 2010 and 2023 (inc)

Met Office. UK and regional series [6.1.25]. Available from: <https://www.metoffice.gov.uk/research/climate/maps-and-data/uk-and-regional-series>.

| **January:** 3.78°C |
| --- |
| **February:** 4.33°C |
| **March:** 5.76°C |
| **April:** 7.88°C |
| **May:** 10.76°C |
| **June:** 13.78°C |
| **July:** 15.57°C |
| **August:** 15.11°C |
| **September:** 13.26°C |
| **October:** 10.21°C |
| **November:** 6.59°C |
| **December:** 4.64°C |

S1: Gout diagnostic codes

| MEDICAL_CODE_ID | DESCRIPTION | READ_CODE | SNOMED_CT_CODE |
| --- | --- | --- | --- |
| 293410018 | Gouty tophi of hand | C34y500 | 190842000 |
| 16455010 | Gouty neuritis | C34y400 | 9386003 |
| 293394013 | Gouty nephropathy | C341.00 | 190829000 |
| 255612014 | O/E - auricle of ear - tophi | 2D52.00 | 164208001 |
| 8.06061E+14 | Gouty tophi of heart | C34y100 | 27277001 |
| 264664010 | Gout monitoring | 669..00 | 170730005 |
| 293407013 | Gouty tophi of other sites | C34y200 | 402469004 |
| 2695828012 | Gouty arthritis of toe | N023800 | 428839004 |
| 359373012 | Drug-induced gout | C344.00 | 239845005 |
| 264675014 | Gout monitoring NOS | 669Z.00 | 170730005 |
| 309679012 | Gouty arthritis of the lower leg | N023600 | 48440001 |
| 309682019 | Gouty arthritis of other specified site | N023y00 | 48440001 |
| 309675018 | Gouty arthritis of the upper arm | N023200 | 201663006 |
| 309683012 | Gouty arthritis NOS | N023z00 | 48440001 |
| 359372019 | Gout due to impairment of renal function | C345.00 | 239844009 |
| 293411019 | Other specified gouty manifestation NOS | C34yz00 | 90560007 |
| 312525018 | [X]Other secondary gout | Nyu1700 | 239843003 |
| 72069017 | Gouty iritis | C34y300 | 43193009 |
| 481958014 | Idiopathic gout | C342.00 | 24595009 |
| 25080011 | Gouty tophi of ear | C34y000 | 14763005 |
| 150085018 | Gout | C34..00 | 90560007 |
| 309674019 | Gouty arthritis of the shoulder region | N023100 | 201663006 |
| 293400010 | Gouty nephropathy NOS | C341z00 | 190829000 |
| 80709010 | Gouty arthritis | N023.00 | 48440001 |
| 309680010 | Gouty arthritis of the ankle and foot | N023700 | 201669005 |
| 309677014 | Gouty arthritis of the hand | N023400 | 201666003 |
| 293392012 | Gouty arthropathy | C340.00 | 190828008 |
| 293412014 | Gout NOS | C34z.00 | 90560007 |
| 309681014 | Gouty arthritis of multiple sites | N023x00 | 201670006 |
| 293405017 | Other specified gouty manifestation | C34y.00 | 3875003 |
| 309676017 | Gouty arthritis of the forearm | N023300 | 48440001 |
| 45604013 | Gouty tophi of heart | G557300 | 27277001 |

Supplementary figure S1: Annual rate of urate testing for each year of the study

Supplementary figure S2: Jointpoint models (detail)

**Model (A): Whole cohort**


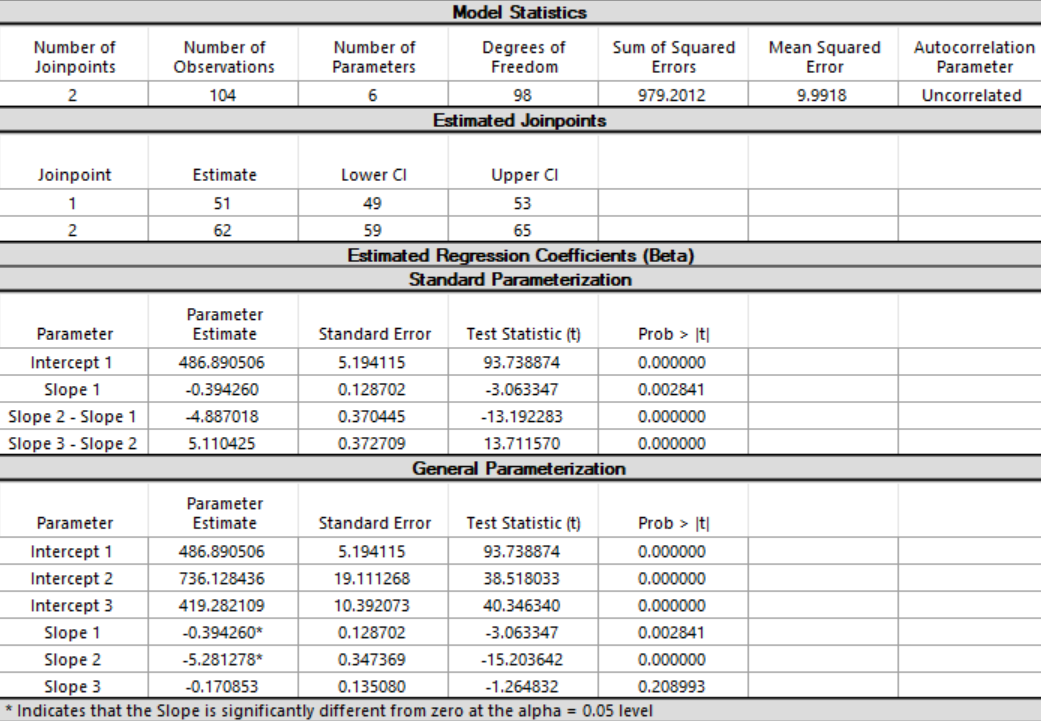


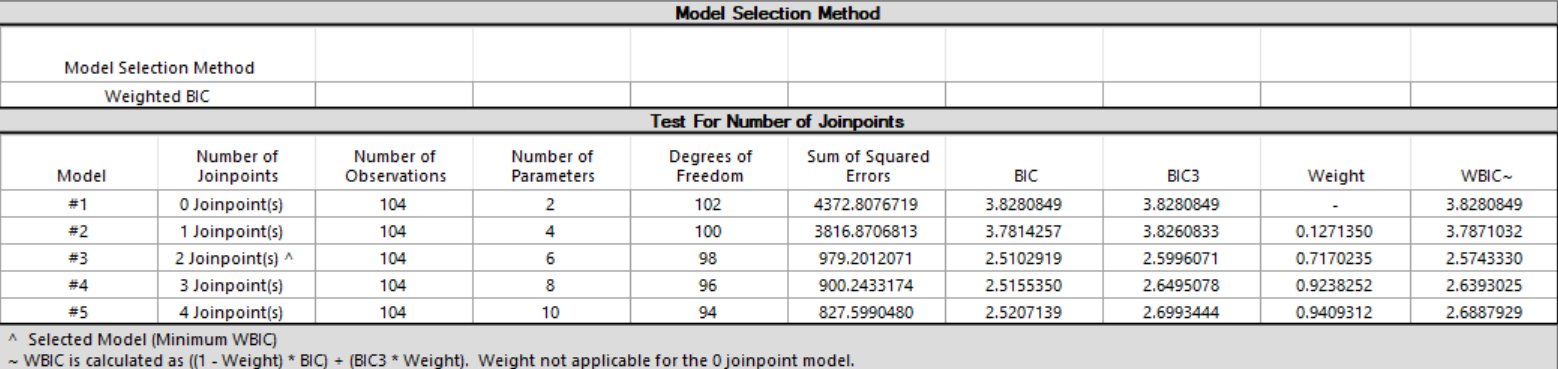


**Model (B): One subsequent flare only**


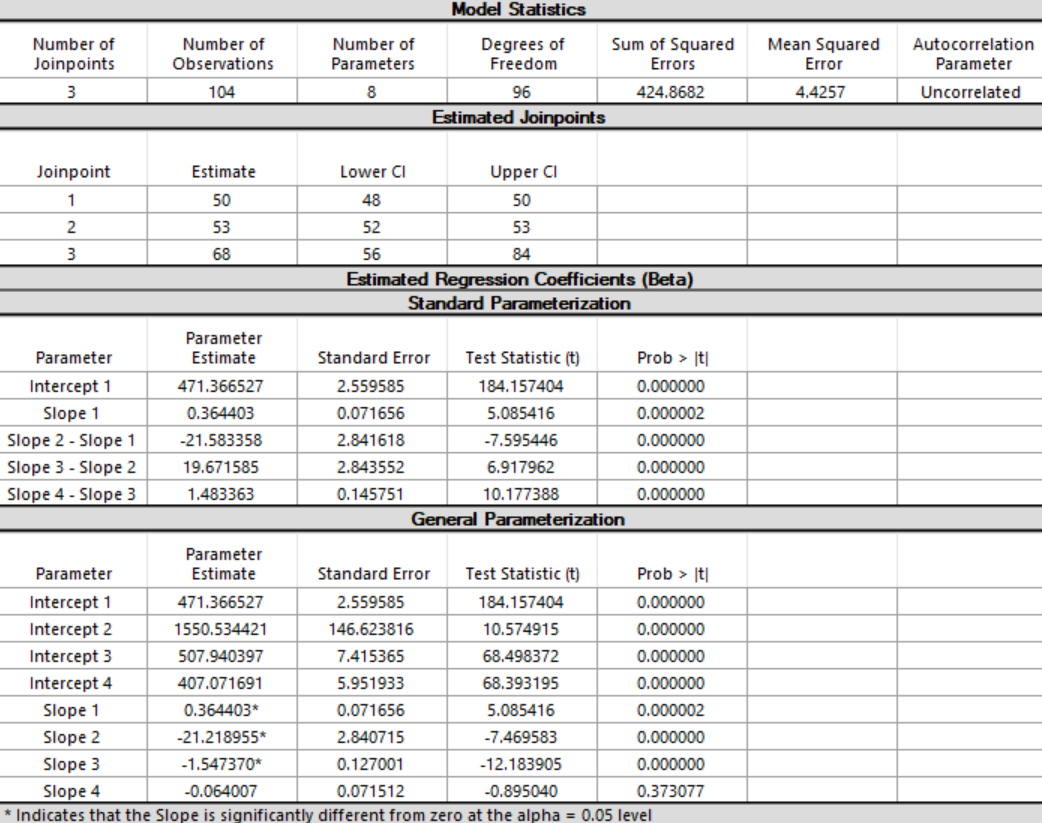


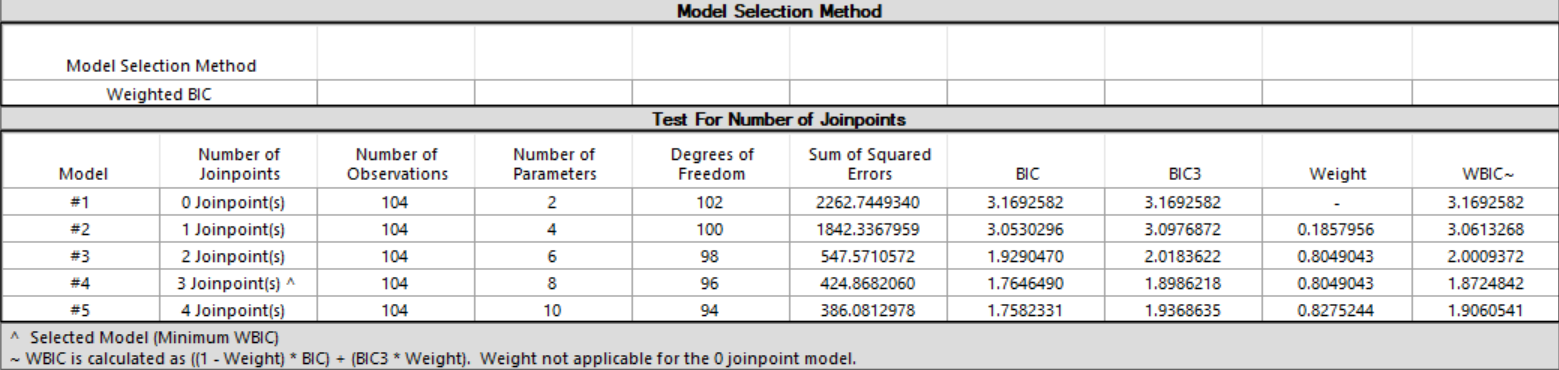


**Model (C): One subsequent flare only *and* where SU available before and after flare**


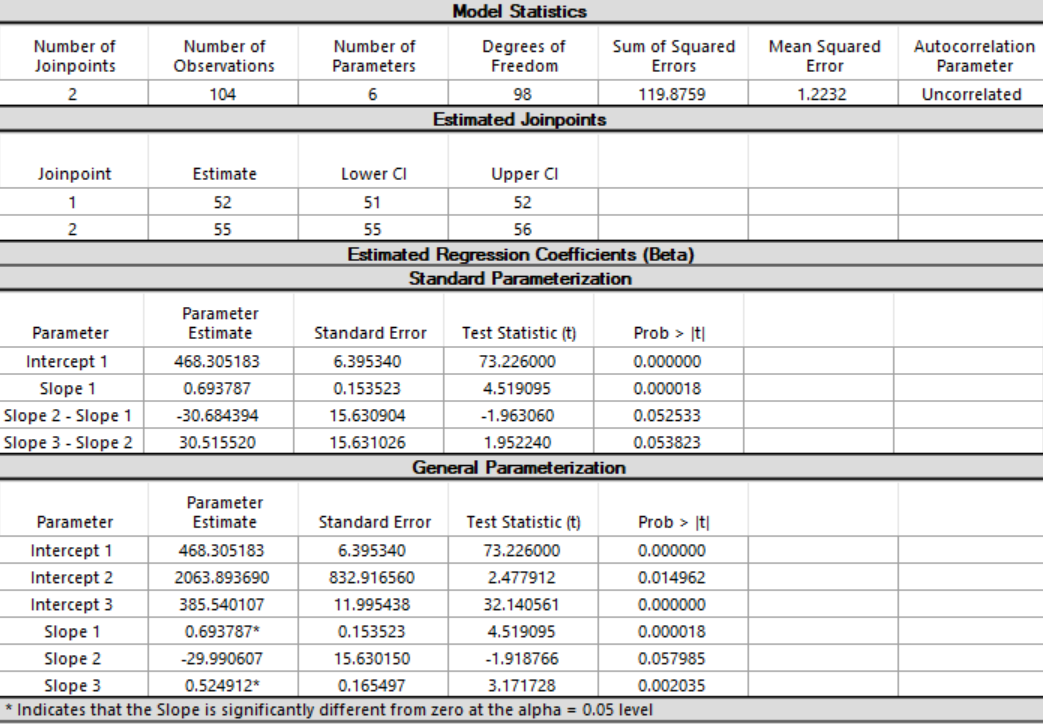


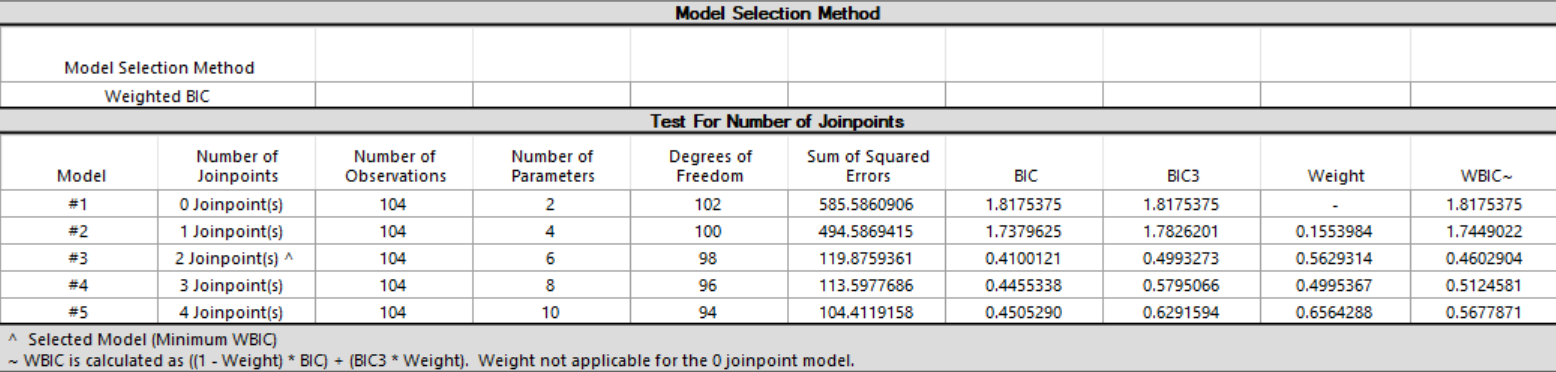


Supplementary figure S3: Relationship between mean monthly SU and flare rate


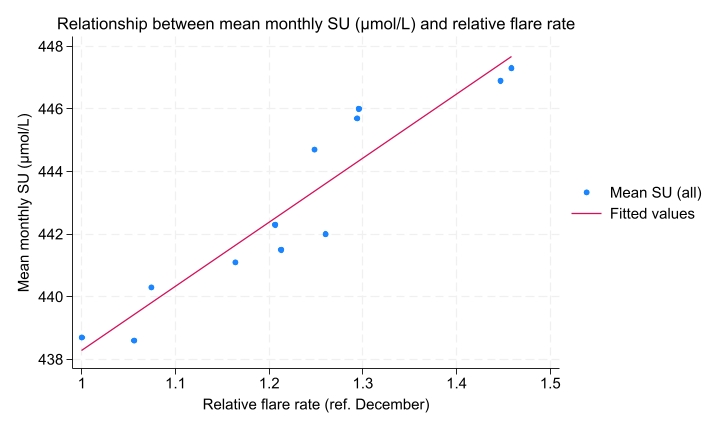

Supplement: Supplementary file 1 — (DOCX 1.09 MB) [file 10067_2025_7898_MOESM1_ESM.docx]
